# Peanuts that keep aflatoxin at bay: a threshold that matters

**DOI:** 10.1111/pbi.12846

**Published:** 2017-10-17

**Authors:** Kiran K. Sharma, Arunima Pothana, Kalyani Prasad, Dilip Shah, Jagdeep Kaur, Deepak Bhatnagar, Zhi‐Yuan Chen, Yenjit Raruang, Jeffrey W. Cary, Kanniah Rajasekaran, Hari Kishan Sudini, Pooja Bhatnagar‐Mathur

**Affiliations:** ^1^ International Crops Research Institute for the Semi‐Arid Tropics (ICRISAT) Hyderabad Telangana India; ^2^ Donald Danforth Plant Science Center St. Louis MO USA; ^3^ Southern Regional Research Center Agricultural Research Service United States Department of Agriculture (USDA/ARS) New Orleans LA USA; ^4^ Department of Plant Pathology and Crop Physiology Agricultural center Louisiana State University Baton Rouge LA USA

**Keywords:** aflatoxin, peanut, *Aspergillus flavus*, food safety, mycotoxins, host‐induced gene silencing, defensins

## Abstract

Aflatoxin contamination in peanuts poses major challenges for vulnerable populations of sub‐Saharan Africa and South Asia. Developing peanut varieties to combat preharvest *Aspergillus flavus* infection and resulting aflatoxin contamination has thus far remained a major challenge, confounded by highly complex peanut–*Aspergilli* pathosystem. Our study reports achieving a high level of resistance in peanut by overexpressing (OE) antifungal plant defensins *MsDef1* and *MtDef4.2*, and through host‐induced gene silencing (HIGS) of *aflM* and *aflP* genes from the aflatoxin biosynthetic pathway. While the former improves genetic resistance to *A. flavus* infection, the latter inhibits aflatoxin production in the event of infection providing durable resistance against different *Aspergillus flavus* morphotype*s* and negligible aflatoxin content in several peanut events/lines well. A strong positive correlation was observed between aflatoxin accumulation and decline in transcription of the aflatoxin biosynthetic pathway genes in both OE‐Def and HIGS lines. Transcriptomic signatures in the resistant lines revealed key mechanisms such as regulation of aflatoxin synthesis, its packaging and export control, besides the role of reactive oxygen species‐scavenging enzymes that render enhanced protection in the OE and HIGS lines. This is the first study to demonstrate highly effective biotechnological strategies for successfully generating peanuts that are near‐immune to aflatoxin contamination, offering a panacea for serious food safety, health and trade issues in the semi‐arid regions.

## Introduction

Aflatoxins, secondary metabolites produced by *Aspergillus flavus* and *A. parasiticus*, are extremely toxic, immunosuppressive and carcinogenic compounds (Bhatnagar‐Mathur *et al*., 2015). Over 5 billion people in developing countries of sub‐Saharan Africa (SSA) and South Asia (SA) are exposed to uncontrolled levels of these toxins, while nearly 2 billion unsuspectingly consume aflatoxins at levels far above the European standards of 4 ppb, especially in low‐income countries where food rarely undergoes formal safety inspection (Shwartzbord and Brown, 2015; Williams *et al*., 2004; Wu, 2014). Alarming levels of aflatoxin contamination in an array of crops including peanuts have been reported around the world (Giorni *et al*., 2007; Jiang *et al*., 2005; Levic *et al*., 2013; Shepherd, 2004; Waliyar *et al*., 2003). Very high levels of aflatoxins B_1_, B_2_, G_1_ and G_2_ in peanuts, peanut butter and other processed commodities sold in formal and informal markets in low‐income countries of SSA and SA are of great concern.

Peanut being a subterranean legume is susceptible to contamination from the soil that serves as a reservoir for *Aspergilli*. The developing peanut pods are in direct contact with soil populations of these two aflatoxigenic species that inhabit soils as conidia or sclerotia. While frequent droughts and high temperatures can cause the pods to shatter, damaging tissues, thereby increasing the chances of preharvest infection, drought adaptation in peanut is not necessarily linked to the level of resistance to *A. flavus* invasion and aflatoxin accumulation (Hamidou *et al*., 2014). Although postharvest management practices such as appropriate drying, curing and storage can minimize aflatoxin contamination during storage, these can be only effective when peanuts are free from preharvest infection. Biocontrol strategies such as ‘competitive atoxigenic’ fungal technology (CAFT) and deploying promiscuous atoxigenic *Aspergillus* strains have been shown to reduce levels of aflatoxin contamination in the field. Nevertheless, CAFT poses potential challenges in peanut, as it does not offer protection from exponential mould growth further compromising peanut quality and hygiene. However, development of varieties with desirable genetic resistance to preharvest infection by *A. flavus* and aflatoxin contamination has remained a challenge for peanut breeding programmes (Bhatnagar‐Mathur *et al*., 2015; Janila and Nigam, 2013).

Here, we describe a host–plant resistance strategy to create peanut germplasm with improved genetic resistance to *A. flavus* infection and aflatoxin contamination. This is performed using a 3‐tier approach involving (i) prevention of fungal infection by boosting the innate plant immunity; (ii) arrest of subsequent fungal growth in the event of infection; and (iii) inhibition of aflatoxin production in scenarios where fungal infection is difficult to eradicate. This approach involved altering interactions of the *Aspergillus*–peanut pathosystem for (i) activation of defence pathways by differentially regulating plant antimicrobial polypeptides (AMPs; defensins) that confer enhanced protection against pathogenic stresses and mechanical wounding (Bhatnagar‐Mathur *et al*., 2015 *;* Goyal and Mattoo, 2014 *;* Kaur *et al*., 2011 *;* Van der Weerden *et al*., 2013) and (ii) expressing double‐stranded RNA molecules of *Aspergillus* in the peanut–host system to inactivate and suppress key aflatoxin biosynthetic pathway genes.

## Results

### Generation and molecular analysis of OE and HIGS lines

**Figure 1:**
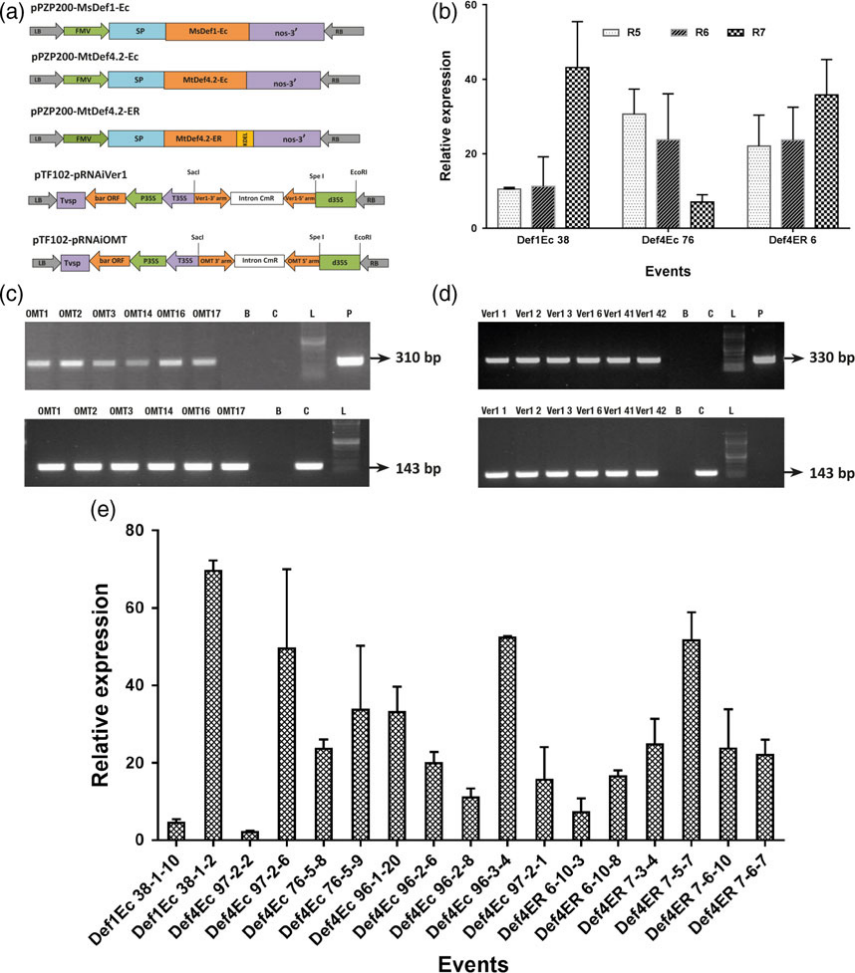
Transformation vectors and expression analysis of peanut OE‐Def and HIGS lines. (a) Expression vectors used for peanut transformation. The constitutive figwort mosaic virus (FMV) 35S promoter was used for expression of full‐length *MsDef1‐Ec*,* MtDef4.2‐Ec* and *MtDef4.2‐ER*. *MsDef1‐Ec* and *MtDef4.2‐EC* constructs targeted each defensin to the apoplast with signal peptide, whereas *MtDef4.2*‐ER construct retained this defensin in the endoplasmic reticulum. For targeting the aflatoxin pathway genes, the hpRNA cassettes had inverted repeats of respective *omtA* (*aflP*) and *ver‐1(aflM)* regions around the PR10 intron under the control of double CaMV 35S promoter. LB, left border; RB, right border; nos, nopaline synthase gene terminator; CaMV35S, cauliflower mosaic virus promoter; SP, signal peptide. (b) Expression of defensin transgenes in various OE‐Def events (pooled across generations) at different pod development stages (R5, R6 and R7). (c,d) RT‐PCR analyses to detect the expression of *hpRNA* transcripts in mature cotyledons. A 310‐bp amplicon for *omtA* (c) and a 330‐bp amplicon for *ver‐1* (d). An intron‐spanning peanut *ADH3* gene was used as a control (lower panel). A 400‐bp amplicon is expected from a genomic DNA template, whereas 143‐bp amplicon is expected from a cDNA template. Letters B and C represent Blank and WT control, respectively; L stands for marker ladder and P denotes plasmid. (e) Expression of defensin transgenes in various OE‐Def events (pooled across generations) in mature cotyledons after infection with *A. flavus* AF11‐4 at 72 hpi. The housekeeping gene, *G6PD* was used for normalization with respect to the WT. Error bars represent the standard error (SE) of at least five replicates.

*Agrobacterium*‐mediated transformation of JL24, a peanut variety susceptible to *Aspergillus flavus* infection and aflatoxin production, resulted in several events that constitutively overexpressed either *MsDef1* and *MtDef4.2*, or the inverted repeat sequences of mid and late aflatoxin biosynthesis genes *ver‐1* (*aflM*) and *omtA* (*aflP*) (Figures 1a and S1). The lines overexpressing defensin genes (OE‐Def) were designed to direct their respective recombinant proteins to the extracellular space (OE‐Def1Ec and OE‐Def4‐Ec) or retained in the endoplasmic reticulum (OE‐Def4‐ER). To identify transgenic events where transgenes segregated as a single locus in T_1_, T_2_ and T_3_ generations, seed progenies were characterized for integration and expression of the transgenes using PCR and qPCR analyses (Tables S1, S4 and S5). From the selected 27 independent transgenic events of peanut for each of the three OE‐Def constructs, 16 independent T_1_ events that showed 3 : 1 segregation of the transgene indicative of a single insert were selfed to obtain homozygous T_2_ and T_3_ progenies. Similarly, from the 12 HIGS lines carrying hairpin RNAs (hpRNAs) that segregated in Mendelian ratios in the T_1_ generation, progenies from eight events were advanced to T_2_ and T_3_ generations. Quantitative real‐time PCR (qRT‐PCR) analyses of these genotypes in T_2_ and T_3_ generations confirmed single transgene integration in most of these events and homozygous progenies were identified. We did not observe any morphological or developmental growth abnormalities in any of these OE‐Def or HIGS lines when compared to the wild type (WT).

The expression of defensin genes was determined during seed filling (R5), full seed (R6) and beginning seed maturity (R7) stages of pod development by qRT‐PCR (Figure 1b). All the selected transgenic plants from both OE‐Def and HIGS lines showed their respective transgene transcription in the mature seeds in RT‐PCR assays (Figures 1c and S2). Further, OE‐Def events maintained a steady transcript abundance (up to 70‐fold) of the respective defensin until 72 h postinoculation (hpi), offering much higher levels of resistance to fungal growth (Figure 1d).

### Challenging assays reveal differential resistance to *A. flavus* infection

**Figure 2:**
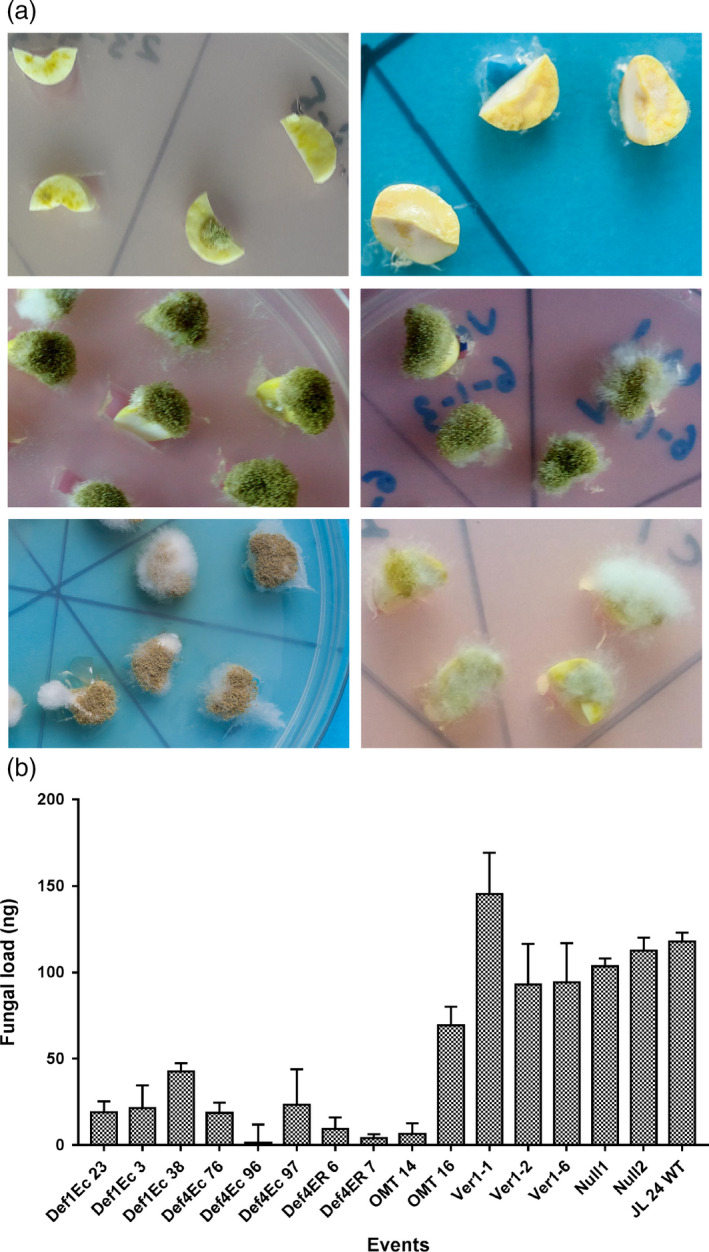
Fungal assay of OE‐Def and HIGS lines at 72 hpi. (a) Comparison of fungal colonization on cotyledons of *MtDef4‐Ec *96 (top row left), *MsDef1‐Ec* 23 (top row right), HIGS line; *hp‐omtA* 16 (middle row left), HIGS lines; *hp‐ver1‐1 6* (middle row right), WT control (last row left) and resistant check, 55‐437 (last row right); OE‐Def lines show no or very little fungal growth on events generated with extracellularly targeted *Def4* and *Def1* genes; HIGS lines show no restriction to fungal growth on events generated with *omtA* and *ver‐1*; extensive fungal growth and sporulation on WT controls, resistant check‐peanut variety 55‐437. (b) Fungal load of *A. flavus* on cotyledons of OE‐Def, HIGS and WT lines after 72 hpi. Error bar represents standard error of at least three biological replicates at *P *=* *0.5.

To test the effectiveness of aflatoxin control in OE‐Def and HIGS lines, fungal bioassays using 5 × 10^4^ spores per mL of *A. flavus* isolate 11‐4 (AF11‐4) were carried out using cotyledons from mature seeds. Fungal invasion, colonization and aflatoxin accumulation were evaluated after 72 hpi. A West African peanut line 55–437 reported as being highly resistant to aflatoxin contamination was also included as a resistant check (RC) in these bioassays. Transgenic peanut plants overexpressing defensins effectively restricted AF11‐4 invasion and colonization very effectively when compared to WT and RC (Figure 2a) in contrast to the HIGS lines that offered very little resistance to the invading fungus (Figure 2a).

Visual observations on peanut cotyledons challenged with AF11‐4 corroborated well with the fungal load measured as fungal biomass on total DNA isolated from the inoculated cotyledons after 72 hpi using qPCR analyses. On an average, 17.2 ng of *A. flavus* DNA was detected in 100 ng of the total DNA sample from OE‐Def events, compared to 81.6 ng and 111.3 ng per 100 ng of the total DNA in the HIGS and Null/WT samples, respectively (Figure 2b). These results revealed that while the inoculated OE‐Def events supported very low fungal load, the infected host tissues of the HIGS lines showed substantial fungal development (Figure 2b). No fungal growth was detected in the un‐inoculated cotyledon samples.

### Significantly reduced aflatoxin accumulation in OE‐Def events and HIGS lines

**Figure 3:**
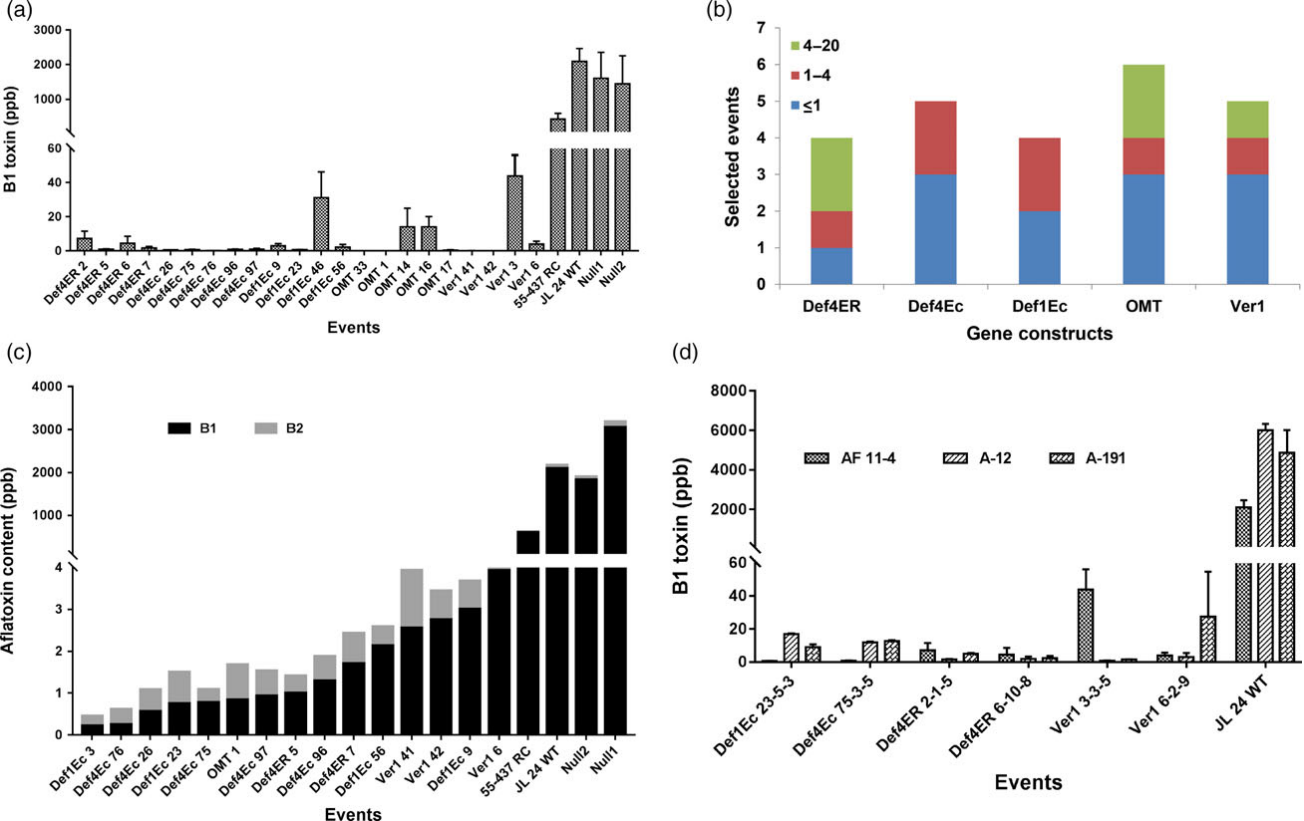
Aflatoxin profile of T_3_ seed cotyledons of OE‐DEf and HIGS peanut lines following *A. flavus* infection at 72 h using HPLC (a) B_1_ levels (ppb) in the inoculated cotyledons of OE‐Def, HIGS and WT peanut lines. (b) Number of best homozygous events across five constructs that accumulate ≤20 ppb B_1_ toxin across 24 selected events. The colour codes reveal the range of B_1_ content based on HPLC. (c) Aflatoxin profiling based on individual toxin types in selected events of OE‐Def and HIGS lines accumulating <4 ppb B_1_ and B_2_ toxins after *A. flavus* (*AF11‐4*) infection. The events were sorted by the content. (d) Event‐wise comparison of B_1_ toxin (ppb) in a subset of homozygous T_3_ progenies of OE‐Def, HIGS and WT peanut lines against three different *A. flavus* morphotypes (AF11‐4, A‐12, A‐191).

The level of aflatoxin B_1_ pooled across 24 selected OE‐Def events and HIGS lines showed a significant reduction of 98.5%–99.0% and 85.0%–99.9%, respectively (at *P *<* *0.01), compared to the inoculated WT controls after 72 hpi with the aflatoxigenic *A. flavus* AF11‐4 (Figure 3a). Several OE‐Def events such as Def4‐Ec26, Def4‐Ec75, Def4‐Ec76, Def4‐Ec96, Def4‐Ec97, Def1‐Ec23, Def4‐ER5 and Def4‐ER7 accumulated <2 ppb B_1_ compared to >2000 ppb, >1200 ppb and >500 ppb detected in the nulls, WT and RC, respectively, thereby indicating very high levels of resistance to aflatoxin contamination. Overall, from the 24 T_2_ and T_3_ events that were tested across seven experiments, six OE‐Def events (three Def4‐Ec, two Def1‐Ec and one Def4‐ER) accumulated <1 ppb B_1_; five events (two each of Def4‐Ec and Def1‐Ec and one of Def4‐ER) accumulated 1–4 ppb B_1_ and two Def4‐ER events accumulated 4–20 ppb B_1_ (Figures 3b, S3 and S4). Similarly, four HIGS lines (three *hp‐ver1* and one *hp‐omtA*) accumulated 1–4 ppb B_1_, and two *hp‐omtA* lines accumulated up to 20 ppb B_1_, all accumulating significantly less (*P *<* *0.01) than the inoculated nulls, WT and RC (Figure 3b). Intriguingly, while the null, WT and RC accumulated minute quantities of G1 and G2 toxins, the OE‐Def and HIGS lines did not accumulate any of these toxins (Figures 3c, S3–S5, Table S2).

Furthermore, *A. flavus* fungal load in the infected cotyledons at 72 hpi when plotted against their respective aflatoxin content showed an unequivocal correlation in the OE‐Def lines (Table S3), demonstrating that defensins confer enhanced resistance to fungal infection and subsequent toxin accumulation. In contrast, a very weak correlation was observed in the HIGS lines where the fungal load had little bearing on aflatoxin biosynthesis (Table S3).

Considering that aflatoxin production by different morphotypes of *A. flavus* varies in peanut, we challenged the seeds of a subset of homozygous T_3_ progenies of OE‐Def and HIGS lines with two other ‘S’ type aflatoxigenic strains (A‐12 and A‐191). Most of the tested peanut lines revealed a significant reduction in the levels of B_1_ toxin ranging from 1 to 20 ppb in comparison with high levels of contamination (>4000 ppb) observed in the WT. Although plant‐to‐plant variability in toxin accumulation was observed with the three different fungal strains, the overall trend was consistent with that obtained using AF11‐4 (Figure 3d).

### ROS homeostasis during host–pathogen interactions

**Figure 4:**
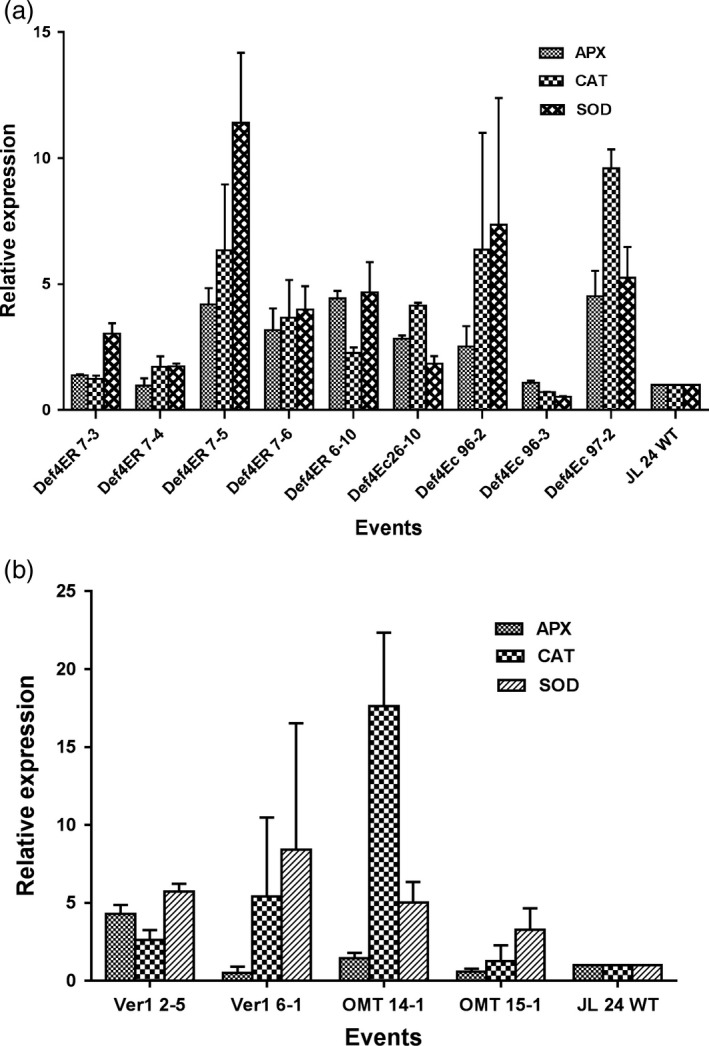
Expression profile of host ROS scavenging antioxidative genes, *SOD*, *CAT* and *APX* in the infected peanut cotyledons of (a) OE‐Def events and (b) HIGS lines in comparison with the WT at 72 hpi.

To gain a mechanistic understanding of resistance to *A. flavus* colonization and aflatoxin contamination in OE‐Def and HIGS lines, we determined the expression of some key peanut genes during host–pathogen interactions. Total RNA from AF11‐4‐infected cotyledons at 72 hpi was used to determine the expression of peanut genes encoding reactive oxygen species (ROS) scavenging enzymes such as *superoxide dismutase* (*SOD*) [Cu–Zn], *ascorbate peroxidase* (*APX*) and *catalase* (*CAT*). In OE‐Def events, the expression of *SOD* [Cu–Zn] increased significantly by 1.7‐ to 11.3‐fold followed by an increase in *CAT* expression by 1.2‐ to 9.5‐fold, and *APX* expression by 1.0‐ to 4.4‐fold when compared to inoculated WT (Figure 4a). This indicated that overexpression of defensins in peanuts provided protection from oxidative damage during fungal infection. Intriguingly, the HIGS lines also demonstrated up‐regulation of *SOD* and *CAT* genes by 3.2‐ to 8.4‐fold and 1.2‐ to 17‐fold, respectively, although no significant changes in the levels of *APX* were observed (Figure 4b). The transcript abundance of *SOD* in both OE‐Def and HIGS lines indicated its role as the first line of defence by converting O_2_ into H_2_O_2_. However, a weaker expression of *APX* in HIGS lines could be an indication of differential modulation of ROS detoxification. These results suggested that both OE‐Def and HIGS lines maintained the ROS homoeostasis possibly through positive regulation of the transcription of *SOD* and *CAT* genes. Nevertheless, no significant correlation was observed between aflatoxin content and expression of host ROS scavenging enzymes in both the types of lines (Table S3).

### Postinfection transcription of aflatoxin synthesis genes

**Figure 5:**
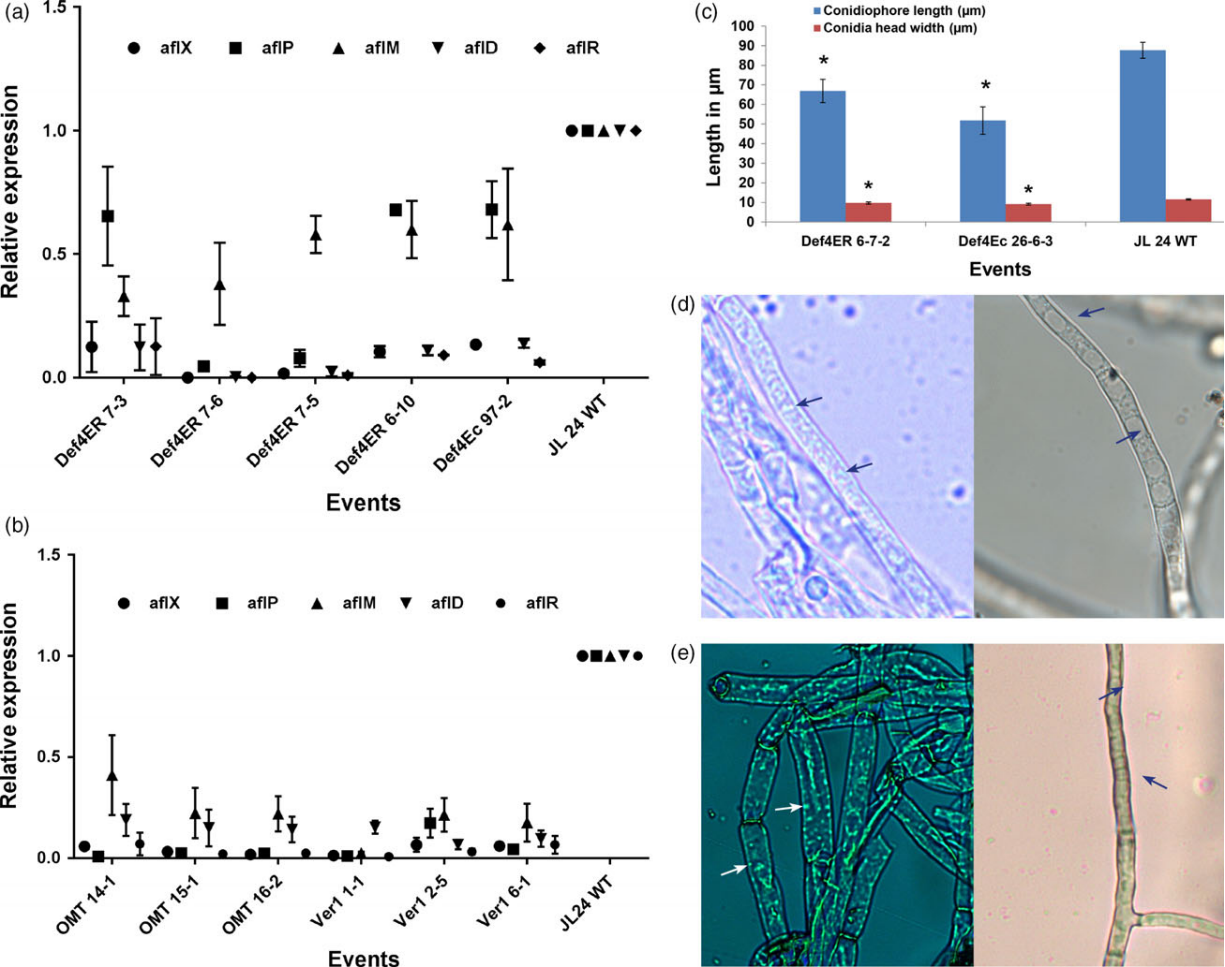
Reduced expression of aflatoxin pathway genes in *A. flavus* and induced morphological alterations in infected transgenic/HIGS peanut lines. (a,b) Transcript abundance of fungal biosynthetic cluster genes in OE‐Def events (a) and (b) HIGS lines in comparison with the WT at 72 hpi. (c–e) Morphology of *A. flavus* (*AF11‐4*) infecting the OE‐Def, HIGS and WT peanut lines after 40  hpi. (c) Conidial morphology of OE‐Def events with WT. (d) Bright‐field microscopy of *A. flavus* at 40 hpi. Profuse vesicles (arrows) detected in the cytoplasm of fungus infecting the WT controls (left) compared to HIGS line OMT15‐1 (right; arrows indicate vacuoles) (magnification at 1000×). (e) High‐intensity staining of vesicles (arrows) reflects higher aflatoxin production ability in the *A. flavus*‐infecting WT (left) compared to HIGS line *Ver1 6‐1* (right; arrows indicate vacuoles) (magnification at 1000×).

To confirm whether the inhibition of aflatoxin biosynthesis observed in our study occurred through gene repression, the relative expression of key early, middle and late aflatoxin biosynthetic pathway genes *aflD*,* aflM*,* aflR aflP* and *aflX* of *A. flavus* at 72 hpi was determined. Significant reductions in transcription of early, middle and late pathway genes were observed in the infected OE‐Def and HIGS lines. The expression levels of *aflX and aflP* in the infected OE‐Def4‐ER and OE‐Def4‐Ec events decreased by 54.0%–99.0% and 22.0%–95.0%, respectively, when compared to the WT. Similarly, the transcripts of *aflM*,* aflD* and *aflR* genes were reduced by 15.0%–79.0%, 67.0%–99.0% and 75.0%–99.9%, respectively, in these OE‐Def events compared to the inoculated WT controls (Figure 5a). While similar results were obtained, the quantum of reduction in gene expression was much higher in most HIGS lines that showed a decrease of 93.4%–98.6% for *aflX*, 82.0%–99.0% for *aflP* and 75.0%–97.0% for *aflM*. The early pathway gene *aflD* showed a reduction of 81.0%–90.0%, whereas the expression of regulatory gene *aflR* was reduced by 81.0%–99.0% (Figure 5b) compared to WT. A strong positive correlation between aflatoxin content and decline in transcription of the aflatoxin biosynthetic pathway genes was observed in both OE‐Def and HIGS lines (Table S3).

### Altered morphology and aflatoxin synthesis/export in the infecting *A. flavus*

Further, histopathological studies in representative OE‐Def samples for *A. flavus* growth and developmental defects during seed infection at 40 hpi were carried out. Transgenic peanut lines overexpressing Def4‐ER6‐7 and Def4‐Ec26‐6‐3 showed a reduction in conidiophore length and conidial head width compared to the WT (Figure 5c). These data indicated that defensins played an important role in defence against *A. flavus* by inhibiting its growth, reducing conidiophore length and alterations in the conidial head width compared with WT. Similarly, the vesicle–vacuole morphology in the very low/negligible aflatoxin accumulating HIGS lines and their WT counterparts was studied after 40 hpi (Figure 5d–e). Following staining with H2CFDA, *A. flavus* hyphae isolated from WT peanut line revealed endosomes predominantly along the cell wall that stained at a higher intensity in contrast to very weak staining observed in the *A. flavus‐*infecting OE‐Def and HIGS lines (Figure 5d,e). These results together with the gene expression data provided sufficient evidence for the existence of a synchronous coordination between aflatoxisome (vesicle) development and the expression of *aflD*,* aflM* and *aflP* genes.

### Trait stability across generations

**Figure 6:**
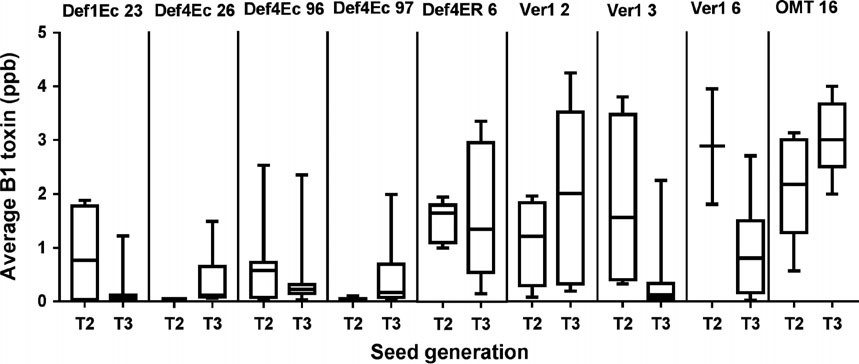
Trait stability in the selected peanut OE‐Def and HIGS lines over three seed generations. The B_1_ content of T2 through T3 seeds remained relatively consistent across selfed generations for lines Def1Ec23, Def4Ec 26, Def4Ec96, Def4Ec97, Def4ER6, Ver‐12, Ver‐13, Ver‐1 6 and OMT16. The box plots show 25%–50% and 50%–75% quartiles (*n *=* *5–13); the mean B_1_ toxin content is shown by the bar.

For successful introgression into elite backgrounds for eventual peanut crop improvement, the trait must be stable across generations. The most promising lines that accumulated little or nondetectable aflatoxin were advanced through single seed descent method (SSD) and selfed. The seeds of progenies from six of these lines assayed for *A. flavus* (AF11‐4) infection and subsequent aflatoxin content revealed high levels of consistency exhibiting trait stability across sexual generations (Figures 6 and S7, Table S6). No differences were observed in agronomic characteristics of these progenies in comparison with the WT. The segregation data indicated true inheritance of defensins and hpRNAs (Tables S4 and S5).

## Discussion

We adopted dual strategies of manipulating the host immunity in peanut by expressing antifungal defensins, *MsDef1* and *MtDef4.2* from *Medicago sativa* and *M. truncatula*, respectively, to confer resistance to preharvest infection, an important step for reducing aflatoxin contamination in peanut and by exploiting the host plant‐induced RNAi silencing of aflatoxin biosynthetic cluster genes (Bhatnagar *et al*., 1991; Cotty and Bhatnagar, 1994) through translocation of sRNA in the invading *A. flavus*.

Higher expression of defensin genes during different stages of pod development in the tested peanut events across all the three gene constructs led us to hypothesize that constitutive expression of these elements of innate immunity might allow a ready host response against fungal pressure and associated stresses, the key factors responsible for preharvest infection and aflatoxin contamination during peanut pod maturation (Koike *et al*., 2002; Mirouze *et al*., 2006; Tamaoki *et al*., 2008).

While the inoculated OE‐Def events had very low fungal load, the infected host tissues of the HIGS lines showed substantial fungal development, which is not surprising as RNAi‐mediated silencing suppresses aflatoxin biosynthesis, and does not affect fungal infection and colonization *per se*. The effectiveness of OE‐Def events in reducing the fungal growth and sporulation strongly indicated inhibition of *A. flavus* growth by *MsDef1* and *MtDef4.2* demonstrating *in planta* antifungal activities of defensins (Gao *et al*., 2000; Kaur *et al*., 2012, 2017; Sagaram *et al*., 2011).

Our choice for targeting *aflM* and *aflP* genes of the aflatoxin biosynthetic pathway was based on data from several previous studies where transcription of these genes in *Aspergillus* coincides with the onset of aflatoxin biosynthesis (Skory *et al*., 1992; Yu *et al*., 1993). These constructs had been previously transformed into corn where a significant reduction in aflatoxin production was reported in a preliminary study (Raruang *et al*., 2016). Peanut RNAi plants targeting early and middle genes of the aflatoxin biosynthesis pathway, as well as genes involved in pathogenesis and aflatoxin efflux, have been shown to accumulate significantly lower aflatoxin in the immature but not in the mature seeds (Arias *et al*., 2015; Power *et al*., 2017). In a recent report, RNAi targeting the *aflC* gene (encoding for *polyketide synthase*,* pksA*) resulted in a significant reduction in the aflatoxin levels (≤93 ppb) in transgenic corn (Thakare *et al*., 2017). However, the reduced aflatoxin levels detected in this study are still way above the 20 ppb aflatoxin limit set by the United States (USDA, 2015) and 2–4 ppb set by the European Union (Codex, 1995).

Several OE‐Def events and HIGS lines demonstrated very high levels of resistance to aflatoxin contamination, accumulating 0.5–20 ppb B_1_ compared to >2000 ppb, >1200 ppb and >500 ppb detected in the nulls, WT and resistant check, respectively. Notably, our data are based on highly stringent phenotyping and event selection using HPLC‐fluorescence quantification, which affords the opportunity to detect as little as 0.1 ppb of aflatoxin in individual mature peanut cotyledons. We did not observe any correlation between aflatoxin content and expression of host ROS scavenging enzymes in both OE‐Def and HIGS lines, inferring that the increased activity of host‐antioxidant enzymes could possibly be an effect of fungal invasion/colonization and not aflatoxin production. Moreover, a strong positive correlation observed between aflatoxin content and decline in transcription of the aflatoxin biosynthetic pathway genes in both OE‐Def and HIGS lines could be attributed to enhanced host resistance to aflatoxin contamination (Alkhayyat and Yu, 2014; Yu, 2012).

Furthermore, as both AFLM and AFLP proteins are synthesized in the fungal cytoplasm and then targeted to specialized vesicles called aflatoxisomes during aflatoxin synthesis, silencing of these genes could have had a significant impact on both upstream and downstream genes such as *aflD, aflR* and *aflX*. This was confirmed by histochemical data that revealed a higher vesicle (aflatoxisome) number in the *A. flavus*‐infecting WT cotyledons, indicative of higher aflatoxin synthesis and its subsequent export outside the fungal cells (Chanda *et al*., 2009). In contrast, a very weak staining of endosomes observed in OE‐Def and HIGS lines might possibly be due to decreased expression of *aflD*,* aflM*,* aflR* and *aflP* genes in the fungus that interfered with the late aflatoxin reactions resulting in greater enzymatic turnover (mRNA decay) in the vacuoles (Chanda *et al*., 2009). The failure to efficiently remove aflatoxin from the fungal cells could possibly also have a feedback inhibitory effect on the transcription of aflatoxin biosynthesis genes that is consistent with the observed decrease in expression of early‐ and mid‐pathway genes such as *aflD* and *aflR* that are positive regulators of aflatoxin biosynthesis (Chanda *et al*., 2009; Yu, 2012).

In summary, our study demonstrated that (i) defensins boosts resistance of peanut against the invading *A. flavus*, providing agronomically useful levels of control, and (ii) functional inhibition of the *ver‐1 (aflM)* and *omtA (aflP)* genes through HIGS results in remarkable resistance to aflatoxin contamination. Our data show that using two different interventions, we achieved aflatoxin levels in peanut that are nondetectable or as low as 1–2 ppb, within the safety limits. This finding is of high significance as there are no resistant peanut lines/varieties available that demonstrate resistance levels even remotely closer to the US or EU legislative limitation of <20 ppb and <4 ppb aflatoxin, respectively. Data presented here suggest that co‐expression of antifungal defensins and hpRNAs targeting mycotoxin genes in transgenic peanuts could boost immunity, potentially resulting in absolute aflatoxin control. As a future follow‐up, we propose a strategy for addressing the complex host–*A. flavus* interactions using biotechnological approaches for effective control of preharvest infection and aflatoxin management in peanut (Figure S8).

## Experimental Procedures

### Vectors and transformations

Defensin genes *MsDef1 and MtDef4.2* were isolated from *Medicago sativa* and *Medicago truncatula*, respectively (Gao *et al*., 2000; Kaur *et al*., 2012). *The* chimeric gene *MsDef1‐Ec* and *MtDef4.2‐Ec* were designed for targeting these defensins to the extracellular space (Kaur *et al*., 2012), while *MtDef4.2‐ER* was used for ER localization. For strong constitutive expression of *Def* genes, 35S promoter from *Figwort mosaic virus* (FMV) and nos terminator were cloned in *Pst*1 site of the binary vector pPZP200 carrying spectinomycin resistance gene for bacterial selection, but devoid of any plant selection marker gene (Bhatnagar *et al*., 2010). HIGS vectors carried synthetic DNA incorporating 310‐bp sections of aflP/omtA (GenBank XM_002379891) and 330 bp of aflM/ver‐1 (GenBank: XM_002379900) cloned as inverted repeats around the PR10 intron as previously described (Chen *et al*., 2010). The double CaMV 35S promoter‐regulated hpRNA cassettes were cloned into pTF102‐PR10‐RNAi vector harbouring the *PAT* gene for resistance to phosphinothricin N‐acetyltransferase and designated as pRNAiOMT and pRNAiVer1, respectively. All four binary vectors were mobilized into *Agrobacterium tumefaciens* strain C58 and used for transformation of peanut variety JL24 using the protocol described earlier (Sharma and Bhatnagar‐Mathur, 2006). For generation advancement and tracking the genetics of each individual event, the embryos were rescued in tissue culture and single seed descent (SSD) was carried out.

### *Aspergillus flavus* growth conditions

*Aspergillus flavus* morphotypes (strains) AF11‐4, A‐12 and A‐191 used in this study were representatives from peanut production systems across different agroecologies in the Indian subcontinent, maintained as collections at ICRISAT (Mehan *et al*., 1995). The aflatoxigenic potential of these strains was confirmed through cultures resulting from two serial single spore transfers. The fungal cultures were grown on potato dextrose broth (PDB) medium at 30 °C in the dark and maintained as 30% glycerol stocks at −80 °C. For inoculum preparation, the fungus was multiplied on soaked and autoclaved peanut seeds to which 5 mL of *A. flavus* spore suspension was added. These were incubated at 28–32 °C for 4–5 days to allow sporulation. Subsequently, the spore suspension of 5 × 10^4^ spores/mL was used for fungal bioassays, where the number of *A. flavus* colonies was counted, and the colony‐forming units (CFUs) determined by standard 10‐fold dilutions to obtain ~40 000 cfu/mL on *Aspergillus flavus parasiticus* agar (AFPA) medium.

### **Bioassays of transgenic peanut with** ***A**. **flavus***

The ability of *A. flavus* to infect transgenic peanut cotyledons was assayed using a reported method (Arias *et al*., 2015) with minor modifications. Briefly, cotyledons of peanut transgenic plants, nulls (segregating progeny without transgene), wild type (WT) and resistant check (RC) were surface sterilized with 0.1% (w/v) aqueous solution of mercuric chloride for 2 min. These were washed thoroughly with sterilized distilled water 2–3× and soaked for 2 h. Subsequently, the seed coats were removed, cotyledons de‐embryonated, cut in half and placed in Petri dishes containing sterile agar (1.5% agar/water; w/v; 12 halved cotyledons per plate), with cut surface exposed. Two microlitres of *A. flavus* spore suspension at the rate of 5 × 10^4 ^spores/mL was used for inoculation, and Petri dishes were incubation at 30 °C in dark. Following 72 h of incubation, inoculated cotyledons were visually observed under 20× magnifying lens for *A. flavus* colonization, mycelial growth and sporulation. Half of each individual seed cotyledons were harvested for aflatoxin measurements, and the other half was used for gene expression studies and fungal load estimations. The samples collected for RNA extraction were immediately frozen in liquid nitrogen and stored in −80 °C until use.

### Aflatoxin analysis

For aflatoxins quantitation, all samples were subjected to ELISA assays and further confirmed using HPLC for accuracy. For this, 100 mg of inoculated cotyledon sample was extracted overnight with 0.5 mL methanol at room temperature (RT) and subsequently filtered through sterile miracloth. Sample extracts were diluted 1 : 10 in PBST‐BSA for quantitative ELISA using standard protocol (Verheecke *et al*., 2014). The selected samples were further analysed for individual toxin types using high‐performance liquid chromatography‐fluorescence detection assay (HPLC). For HPLC estimations, the samples were further reconstituted to 4 mL volume using HPLC grade methanol. Twenty millilitre of phosphate buffered saline (PBS) was added to the 4 mL methanol extract, and sample was cleaned up using an immunoaffinity column (FLAPREP®–R‐Biopharm, Darmstadt, Germany). Samples were eluted with 1 mL methanol and enriched by solvent elimination using RTurbovap and concentrated to 0.3 mL. Forty μL of this eluted sample was injected for quantification on HPLC‐fluorescence detection (HPLC; Waters–Model‐2695; Fluorescence detector – Model – 2475, Waters‐ India) with KOBRA cell for derivatization. The limit of detection attained with this system was 0.1 ng/mL. Subsequently, the concentration of aflatoxins in the tissue samples was calculated against the calibration curve plotted using series of reference standards for B_1_, B_2_, G_1_ and G_2_ (Sigma‐Aldrich, St. Louis, MO) and expressed in ng/g of dry seed weight.

### DNA isolation

Fungal genomic DNA was isolated using 100 mg of mycelium from *A. flavus* cultures using PureLink Plant Total DNA Purification kit (Invitrogen, Carlsbad, CA, USA). The purified DNA was evaluated in 0.8% (w/v) agarose gel followed by quantitative and qualitative determination using Qubit^®^ Fluorometer 2.0 and spectrophotometer (GE Healthcare, New Jersey, USA), respectively, and stored at −20 °C until use.

The plant genomic DNA was extracted from 1 g leaf samples from 30‐day‐old transgenic and wild‐type (WT) peanut using a standard protocol (Dellaporta *et al*., 1983) and quantified using NanoVue Plus™ (GE Healthcare). For the estimation of fungal load in the host tissues, genomic DNA from healthy and infected peanut cotyledon samples was isolated using NucleoSpin plant II midi kit (Macherey‐Nagel, Duren, Germany) following the manufacturer's protocol.

### Fungal load detection

For fungal biomass detection in the host tissues, qPCR assay was conducted. Standard curve ranging from 10 ng to 0.01 pg *A. flavus* DNA was used. *A. flavus* ITS2 region using a pair of FLAV sequence‐specific primers (Sardinas *et al*., 2011) was amplified for qPCR. Cycling conditions used were 95 °C for 3 min, 40 cycles of 95 °C for 10 s and 60 °C for 30 s (during which the fluorescence was measured). The logarithm of starting quantity of template for each dilution was plotted against the cycle threshold values (Ct) to obtain the standard curve (Figure S6). Amplification efficiencies were calculated from the slopes of the standard curves (Kubista *et al*., 2006).

### RNA isolation and library preparation

Total RNA was isolated using RNeasy^®^ Plant Mini Kit (Qiangen, GmbH, Hilden Germany), according to the manufacturer's protocol. The purity and concentration of the isolated RNA were determined using gel electrophoresis and NanoVue plus spectrophotometer (GE Healthcare), diluted to 100 ng/μL for use in RT‐PCR and qRT‐PCR studies. The isolated RNA was tested for DNA contamination in PCR using ADH3 spanning intron primers (Table S1).

### Nucleic acids detection and expression analyses

Polymerase chain reaction (PCR) analysis of the genomic DNA was carried out using gene‐specific primers (Table S1) to detect transgene integration. PCR was performed in 10 μL volume comprising of 5 μL of Emerald Amp^R^ PCR Master Mix, 1 μL of genomic DNA (100 ng), 0.25 μL each of forward and reverse primers (25 pM). PCRs were performed in an Eppendorf thermal cycler (Eppendorf, Germany). PCR amplification profile included denaturation at 95 °C for 5 min; followed by 36 cycles of 95 °C for 60 s, 56 °C (Table S1) for 60 s, 72 °C for 1 min; and a final extension at 72 °C for 10 min. The PCR products were resolved on a 1.2% agarose gel, visualized and documented.

To study the expression of defensin genes in segregating populations, RT‐PCR analysis was carried out using specific primer pairs (Table S1) using the Thermoscript RT‐PCR system (Invitrogen) for cDNA synthesis. Quantitative PCR (qPCR) analyses were carried out with gene‐specific primers (Table S1) in a Realplex PCR System (Eppendorf) using 1 : 3 dilutions of cDNA, 2× SensiFAST™ (Bioline, UK), and 400 nm of each primer in a total volume of 10 μL. The reactions were denatured at 95 °C for 3 min followed by 40 cycles of denaturation at 95 °C for 10 s, and annealing at 60 °C for 30 s. Dissociation curves were performed for each reaction run. A stable peanut housekeeping gene, *glucose‐6‐phosphate dehydrogenase*,* G6Pd* (Reddy *et al*., 2013) was used as an internal reference for transgene expression. For the expression analyses of fungal infected peanut cotyledon samples, *G6PD* and *ß‐tubulin* reference genes from plant and *A. flavus,* respectively, were used (Table S1). Data analyses were carried out using 2‐ΔΔCT (Livak and Schmittgen, 2001), and fold change differences were expressed as Log2 of the number of cycles.

### Copy number detection

Real‐time qPCR assays for copy number detection were designed against FMV promoter sequences for OE‐Def events and PR10 in HIGS lines. Low copy number genes from peanut such as vacuolar protein sorting‐associated protein 53 A‐like (GnVP, Gene ID 107638771) and alcohol dehydrogenase class‐3 (ADH3, Gene ID 107647857) were used as references genes for copy number detection. The standard curves for both the reference gene (GnVP) and transgene (FMV) were generated using genomic DNA dilutions. For unknown samples, 10 ng of genomic DNA was used for copy number detection by qPCR in Realplex (Eppendorf) Real Time PCR system using 2× SensiFAST™ SYBR No‐ROX (Bioline) kit. Three biological replicates per event were analysed, including WT samples and no template controls.

The standard curves were generated and different parameters, for example efficiency (*E*), correlation coefficient (*R* ^2^), slope (*S*) and *y*‐axis intercept of the curve and other parameters were analysed through Eppendorf Mastercycler®ep realplex software. The technical replicates showing a Ct standard deviation of <0.3 and standard curves having *R* ^2^ value of >0.95 were chosen. Transgene copy numbers were estimated using equation X_0_/R_0_ = 10^[(Ct^_X_^−I^_X_^)/S^_X_^] − [(Ct^_R_^− I^_R_^)/S^_R_^]^ (Weng *et al*., 2004), where *I*_X_ and *I*_R_ are intercepts of the relative standard curves, and *S*_X_ and *S*_R_ are slopes of the standard curves of target and reference genes, respectively. Ct_X_ and Ct_R_ are the detected threshold cycles of the amplification of the target and reference genes of the unknown test sample. The copy number of both reference genes (Ro) for both GnVp and ADH3 was taken as 2 copies in the tetraploid peanut genome (based on Southern blot analysis and peanut genome database) for copy number estimation. Sequences for target and reference assays are in Table S1.

### Microscopic studies

Mycelia from infected cotyledons were collected at different time points on glass slides and incubated for 10–15 min with H2DCFDA (Molecular Probes, Invitrogen, USA), covered with coverslip and allowed to stand for 15 min in dark at RT prior to visualization of vacuoles and vesicles at 550 nm using Olympus BX51 microscope (Olympus America Inc., Pennsylvania). Images were captured using a Retiga 2000R camera (Qimaging, Surrey, Canada) and processed with QCaptureVer. 3.1.1. Mycelial fragments were analysed in 10 fields under a light microscope at 40 hpi to measure conidiation, length of conidiophores and conidial head width as mean values of 10‐12 individual measurements at 40× magnification using scale bar 50 μm.

### Statistical analysis

Statistical analyses were performed with SAS (version 9.1, SAS Institute Inc., Cary, NC, USA) using the analysis of variance (ANOVA) in conjunction with a Tukey's multiple comparison test using a *P*‐value of *P *<* *0.05 for samples that were significantly different. All data were presented as means ± standard error (SE) of at least three biological replicates. Means displaying nonmatching lowercase letters were significantly different. Differences between test samples and controls within the same time of sampling were performed using two‐way ANOVA and considered to be significant at *P ≤* *0.05; **P ≤* *0.01; ***P ≤* *.001. The correlations between fungal load and aflatoxin content were determined using Pearson's correlation (*asterisks indicate statistically significant differences at *P *≤* *0.05).

## Supporting information


**Figure S1** The aflatoxin biosynthesis pathway for B_1_ and B_2_, toxin depicting the enzymatic steps (bold and *) targeted for host induced gene silencing (HIGS).
**Figure S2** RT‐PCR analysis of total RNA from mature seeds of OE‐Def events.
**Figure S3** Chromatograms of representative peanut OE‐Def events, depicting peaks for aflatoxins (B_1_, B_2_, G_1_ and G_2_) in OE‐Def4‐Ec 96 (a), and OE‐Def4‐Ec 97 (b), resistant check, 55‐437 (c) and WT control (d).
**Figure S4** Chromatograms of representative peanut OE‐Def events, depicting peaks for aflatoxins (B_1_, B_2_, G_1_ and G_2_) in OE‐Def4‐ER 6 (a), OE‐Def1‐Ec 23 (b) and WT control (c).
**Figure S5** Chromatograms of representative peanut HIGS lines, depicting peaks for aflatoxins (B_1_, B_2_, G_1_ and G_2_) in hp‐omtA 16 (a), hp‐ver1‐6 (b), and WT control (c).
**Figure S6** Calibration of qPCR for quantification of *A. flavus* strain 11‐4 in infected host tissues.
**Figure S7** Progeny analyses of low aflatoxin accumulating peanut OE‐Def and HIGS lines.
**Figure S8** Proposed strategy for tackling the complexities of *A. flavus‐peanut* pathosystem and aflatoxin accumulation.
**Table S1** List of oligonucleotide primers used in this study.
**Table S2** Individual toxin quantification on peanut cotyledons after *A. flavus* (AF11‐4) infection.
**Table S3** Linear regression analysis between accumulated aflatoxin versus fungal colonization, gene expression of host antioxidative machinery and *A. flavus* aflatoxin cluster genes in OE‐Def and HIGS peanut lines.
**Table S4** Inheritance studies in segregating peanut OE‐Def events during T_1_, T_2_ and T_3_ generations.
**Table S5** Inheritance studies in segregating peanut HIGS lines during T_1_, T_2_ and T_3_ generations.
**Table S6** Overall list of selected lines used in the study and analyses applied to each throughout.
