# Supplementary material for: Peanuts that keep aflatoxin at bay: a threshold that matters
Source: Plant Biotechnol J. 2017 Oct 17;16(5):1024–33. doi: 10.1111/pbi.12846 (PMC5902767; doi:10.1111/pbi.12846)
Supplement: Supplementary file 1 — Figure S1 The aflatoxin biosynthesis pathway for B1 and B2, toxin depicting the enzymatic steps (bold and *) targeted for host induced gene silencing (HIGS). Figure S2 RT‐PCR analysis of total RNA from mature seeds of OE‐Def events. Figure S3 Chromatograms of representative peanut OE‐Def events, depicting peaks for aflatoxins (B1, B2, G1 and G2) in OE‐Def4‐Ec 96 (a), and OE‐Def4‐Ec 97 (b), resistant check, 55‐437 (c) and WT control (d). Figure S4 Chromatograms of representative peanut OE‐Def events, depicting peaks for aflatoxins (B1, B2, G1 and G2) in OE‐Def4‐ER 6 (a), OE‐Def1‐Ec 23 (b) and WT control (c). Figure S5 Chromatograms of representative peanut HIGS lines, depicting peaks for aflatoxins (B1, B2, G1 and G2) in hp‐omtA 16 (a), hp‐ver1‐6 (b), and WT control (c). Figure S6 Calibration of qPCR for quantification of A. flavus strain 11‐4 in infected host tissues. Figure S7 Progeny analyses of low aflatoxin accumulating peanut OE‐Def and HIGS lines. Figure S8 Proposed strategy for tackling the complexities of A. flavus‐peanut pathosystem and aflatoxin accumulation. Table S1 List of oligonucleotide primers used in this study. Table S2 Individual toxin quantification on peanut cotyledons after A. flavus (AF11‐4) infection. Table S3 Linear regression analysis between accumulated aflatoxin versus fungal colonization, gene expression of host antioxidative machinery and A. flavus aflatoxin cluster genes in OE‐Def and HIGS peanut lines. Table S4 Inheritance studies in segregating peanut OE‐Def events during T1, T2 and T3 generations. Table S5 Inheritance studies in segregating peanut HIGS lines during T1, T2 and T3 generations. Table S6 Overall list of selected lines used in the study and analyses applied to each throughout. [file PBI-16-1024-s001.pdf]

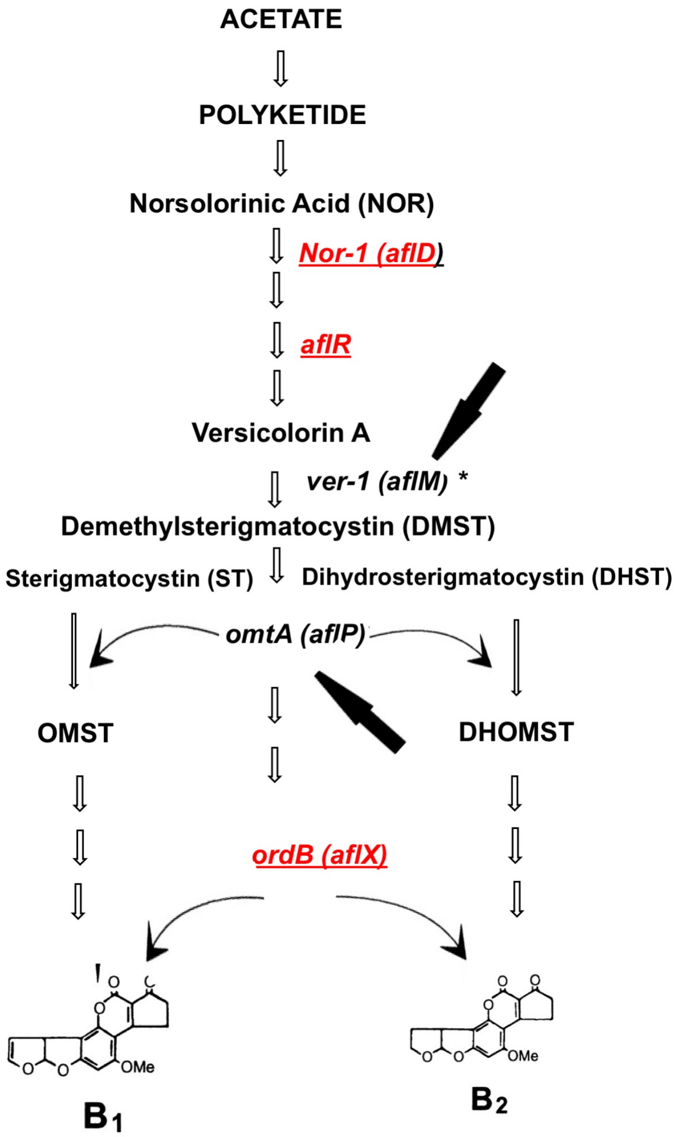

Fig. S1. The aflatoxin biosynthesis pathway for B_1 and B_2, toxin depicting the enzymatic steps (bold and *) targeted for host induced gene silencing (HIGS). The pathway genes studied for changes in transcript expression in *A. flavus* infecting the peanut cotyledons are given in red, italicized and underlined.

Figure S2

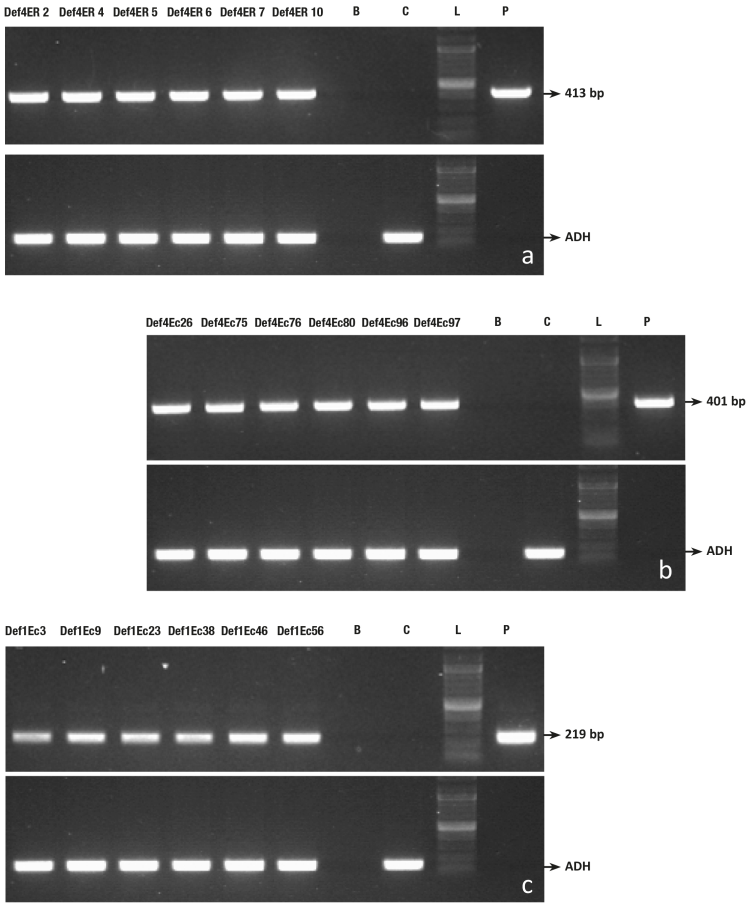

Fig. S2. RT-PCR analysis of total RNA from mature seeds of OE-Def events (A) cDNA from plants transformed with each of OE-Def constructs (OE-Def4-ER, OE-Def4-Ec, and OE-Def1-Ec) and WT control was used to amplify the inserted transgene with amplicon sizes of 413 bp, 401 bp and 219 bp, respectively (upper panel) and an endogenous gene (*ADH3*; lower panel). The presence of genomic DNA in the cDNA preparation would be detected with the *ADH3* primers, which produces a 450 bp band instead of 143 bp (not seen in any of the samples).

Figure S3 a-d

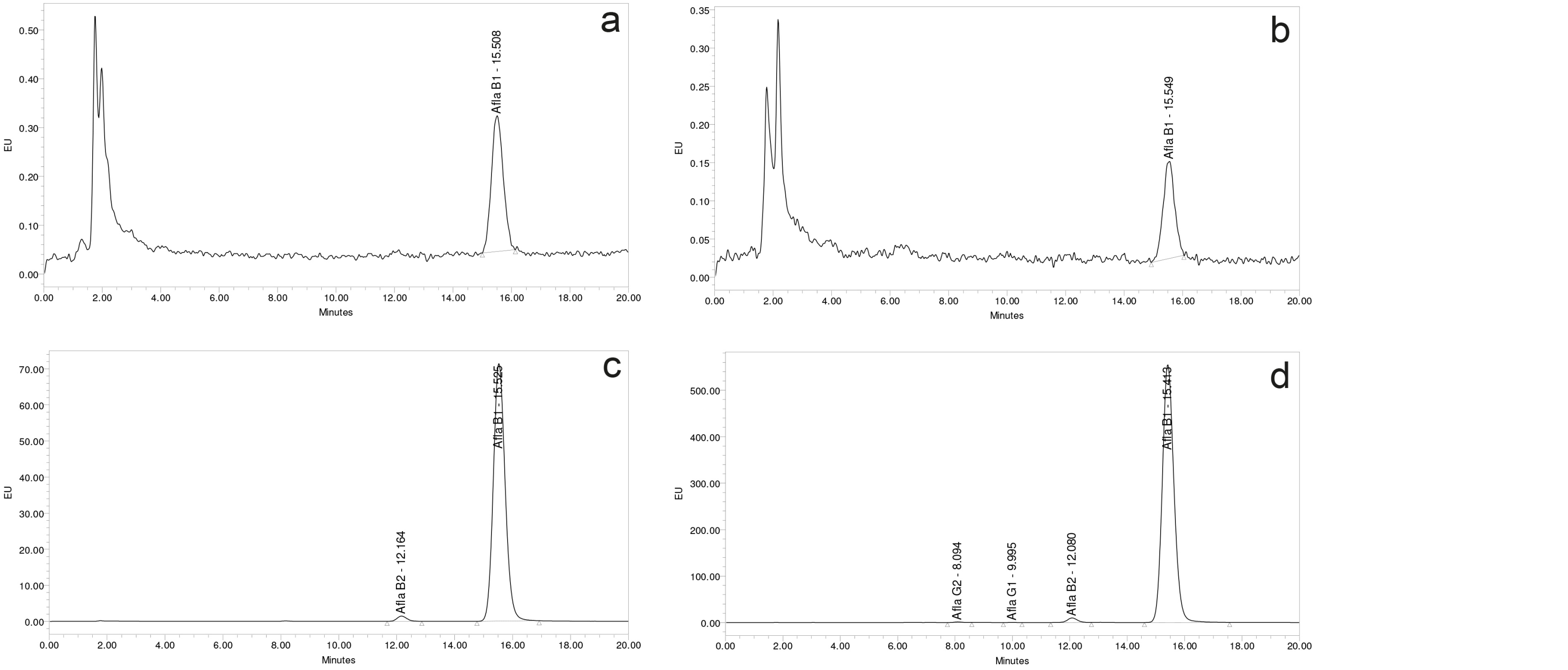

Fig. S3. Chromatograms of representative peanut events depicting peaks for aflatoxins (B_1, B_2, G_1 and G_2) in OE-Def4-Ec 96 (a), and OE-Def4-Ec 97 (b), resistant check, 55-437 (c) and WT control (d).

Figure S4 a-c

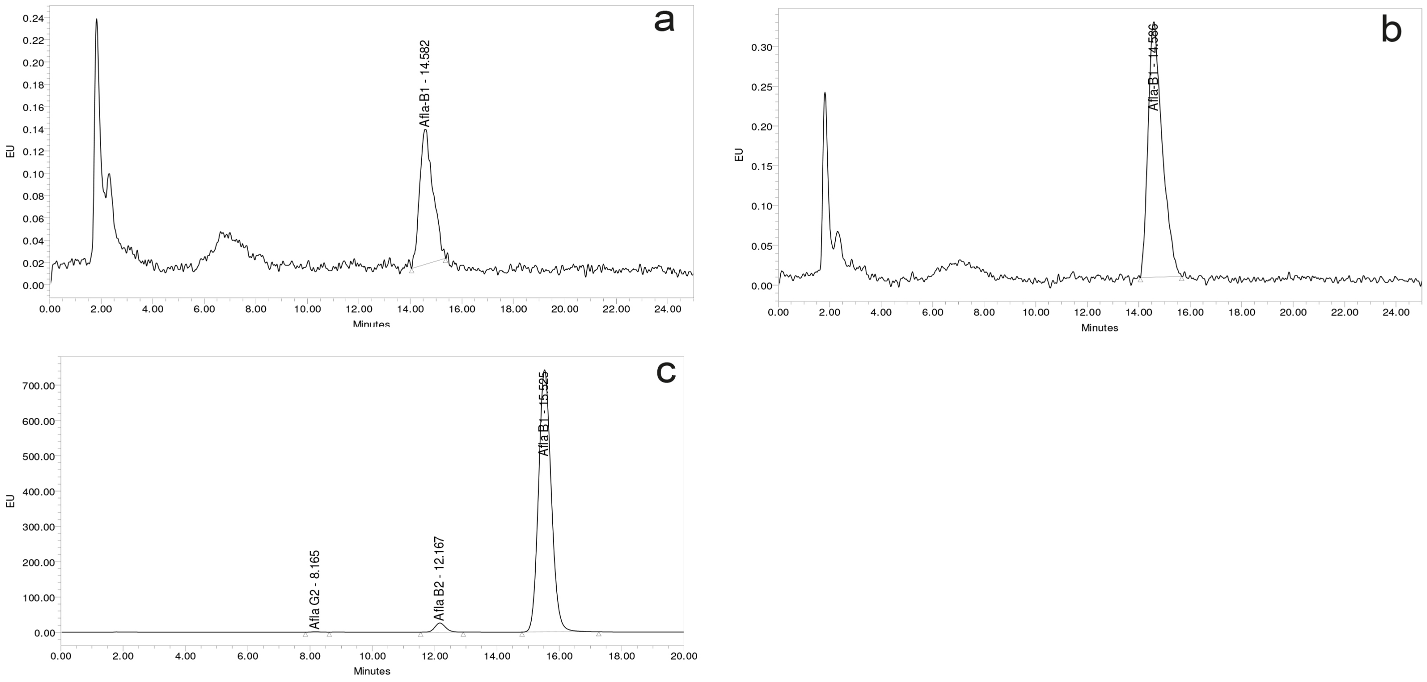

Fig. S4. Chromatograms of representative peanut OE-Def events, depicting peaks for aflatoxins (B_1, B_2, G_1 and G_2) in OE-Def4-ER 6 (a), OE-Def1-Ec 23 (b) and WT control (c).

Figure S5 a-c

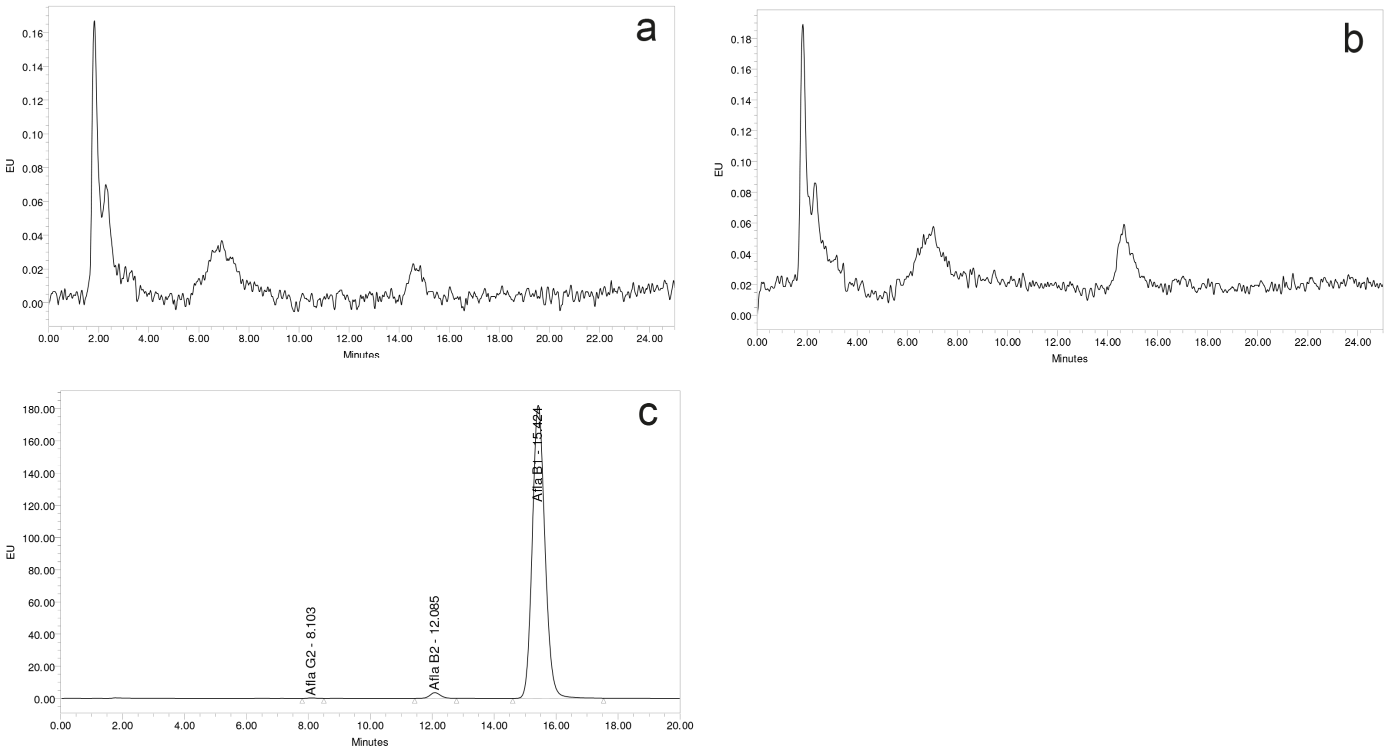

Fig. S5. Chromatograms of representative peanut HIGS lines, depicting peaks for aflatoxins (B_1, B_2, G_1 and G_2) in hp-omtA 16 (a), hp-ver1-6 (b), and WT control (c).

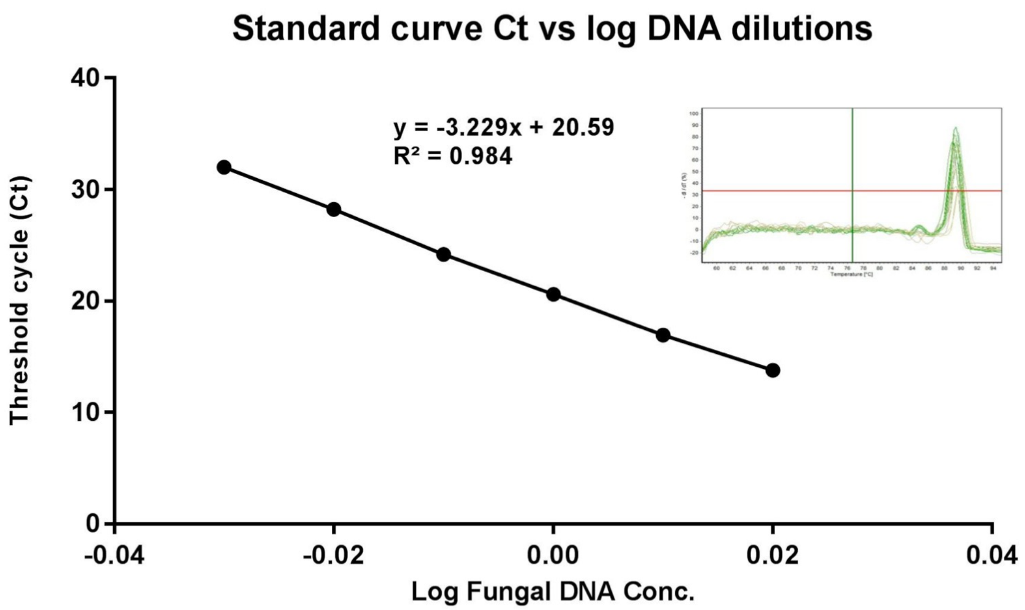

Fig. S6. Calibration of qPCR for quantification of *A. flavus* strain 11-4 in infected host **tissues.** (a) *Standard curve analysis*: Standard curve showing the correlation between the log^{10} DNA amounts (ng) vs. the Ct values for 10-fold dilution of pure fungal genomic DNA. (b) *Melting curve analysis*: The melting curve (SYBR Green florescence versus temperature) of specific amplifications from ITS2 region at different concentrations. No contaminating product was detected in PCR reactions.

Fig. S7. Progeny analyses of short listed low aflatoxin accumulating peanut OE-Def and HIGS lines. The segregation of selected homozygous progeny is highlighted in bold. Events with single copy insert are shown with *, with ** indicating 2 copies (based on qPCR data). The identified best bets based on stable genetics and aflatoxin trait have been advanced (highlighted and italicized).

Primary Transformant	T1	T2	T3	T4
Def1Ec 3	(8:1)	**3-3 →(5:0)**		
		**3-6 →(4:0)**	***3-6 (1-4)***	
		**3-7 →(1:0)**	***3-7 (1)***	
		**3-10 → (2:0)**		
Def1Ec 23	(5:3)	23-5* → (7:1)	**23-5-2 →(10:0)**	***23-5-2 (1-10)***
			**26-6-3→ (6:0)**	***26-6-3 (1-6)***
Def4Ec 26	(6:4)	26-6 → (5:1)		
			26-6-4 →(3:2)	
		**26-10 →(2:0)**	***26-10 (1-2)***	
			80-1-6 →(3:1)	
Def4Ec 80	(5:3)	80-1 → (12:1)	**80-1-8→(4:0)**	***80-1-8 (1-4)***
			80-1-10→(2:2)	
			96-1-1 →(3:3)	
Def4Ec 96	(5:0)	96-1 → (15:5)		
			96-1-10 →(4:2)	
		**96-2 →(10:0)**	***96-2 (1-10)***	
			97-1-3→ (5:1)	
Def4Ec 97	(5:2)	97-1 →(10:2)		
			97-1-11 →(4:1)	
		**97-2* → (7:0)**	***97-2 (1-7)***	
			6-7-2 →(5:1)	
Def4ER 6	(8:2)	6-7 → (9:1)		
			6-7-10 →(4:2)	
		6-10 → (8:4)	**6-10-8→ (6:0)**	***6-10-8 (1-6)***
Def4ER 7	(8:0)	7-5** →(11:0)	***7-5 (1-11)***	
		**7-6 → (17:0)**	***7-6 (1-17)***	
OMT 1	(5:0)	1-1 → (10:3)	1-1-11*→ (8:4)	
OMT 14	(2:0)	**14-1* → (11:0)**	***14-1 (1-11)***	
			16-1-2 →(5:1)	
OMT 16	(2:0)	16-1 → (9:6)		
			**16-1-15→ (2:0)**	***16-1-15 (1-2)***
			2-3-1→ (4:2)	
Ver1 2	(6:2)	2-3→ (9:6)		
			2-3-8→ (5:1)	
Ver1 3	(6:1)	3-2 → (10:4)		
			**3-3-5 →(8:0)**	***3-3-5 (1-8)***
		3-3 → (8:2)	3-3-7 (8:2)	
			**3-3-8 →(4:0)**	***3-3-8 (1-4)***
Ver1 6	(2:0)	6-1 →(6:4)		
			6-2-4*→ (10:1)	
		6-2 →(6:2)		
			**6-2-9→ (11:0)**	***6-2-9 (1-11)***

Fig. S8. Proposed strategy for tackling the complexities of *A. flavus-peanut* pathosystem and aflatoxin accumulation.

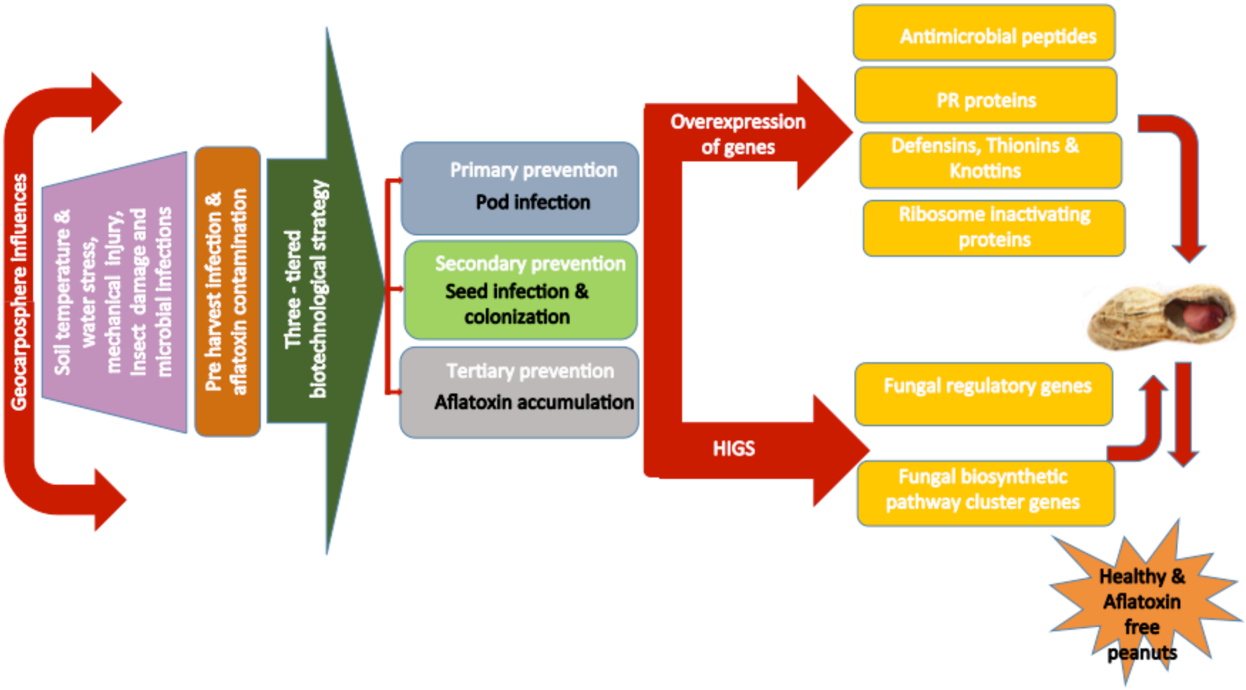

Table S1. List of oligonucleotide primers used in this study.

S. No.	Primer code	Primer Sequence	Tm (°C)	Primer used for	Product Size
1	AhAPXF	TGCTGGAACTTTTGATGTGG	60	qRT-PCR	167 bp
	AhAPXR	AACTACACCGGCCAACTG			
2	AhCATF	CTAGGGGAGCAAGTGCAAAG	60	qRT-PCR	194 bp
	AhCATR	CTCCCGGGTGTAAAACTTCA			
3	AhSODF	CAGTTCTTAGCAGCAGTGAG	60	qRT-PCR	118 bp
	AhSODR	GGAACCCATGAAGACCAG			
4	MsDef1Ec F	GCACAAGAAATTGTGGTGACA	60	qRT-PCR	153 bp
	MsDef1Ec R	AGTACACCAGCAGCGGAAGT			
5	MtDef4 F	TTCCTTTGGTTTCCACCATC	60	qRT-PCR	244 bp
	MtDef4 R	TGCTTCTGCCACCATACTTG			
3	aflR F	GAAGACAGGGTGCTTTGCTC	60	qRT-PCR	225 bp
	aflR R	CTCAACGCCTCATGCTCATA			
4	aflD F	GTGGTGGTTGCCAATGCG	60	qRT-PCR	116 bp
	aflD R	CTGAAACAGTAGGACGGGAGC			
5	aflJ F	ATAAAGTCAGCGGCGTGGTG	60	qRT-PCR	307 bp
	aflJ R	ATGACCGGCACCTTAGCAGT			
6	aflKF	GAGCGACAGGAGTAACCGTAAG	60	qRT-PCR	120 bp
	aflKR	CCGATTCCAGACACCATTAGCA			
7	aflM F	AGCCAAAGTCGTGGTGAACT	60	qRT-PCR	215 bp
	aflM R	CTTTCAGGTGACCGAACGAT			
8	aflO F	GATTGGGATGTGGTCATGCGATT	60	qRT-PCR	181 bp
	aflO R	GCCTGGGTCCGAAGAATGC			
9	aflP F	CACTCGACACAATCGTCACC	60	qRT-PCR	109 bp
	aflP R	TATTGATCAGGGGCAACCTC			
10	aflX F	AGGTTTGACGAAGGTTGTGG	60	qRT-PCR	216 bp

	aflX R	GCACTGAAAAAGCTCCAAGC			
11	ß-tubulin F	GCCGCTTTTTGACTTGCTCC	60	qRT-PCR	231 bp
	ß-tubulin R	ACTGATTGCCGATACGCTGG			
12	MsDef1Ec F	ATGGAGAAGAAATCACTAGCTGGC	56	PCR & RT-PCR	219 bp
	MsDef1Ec R	TTAACATCTTTTAGTACACCAGCA			
13	MtDef4ER F	ATGGCTCGTTCAGTTCCTTTGG	56	PCR & RT-PCR	413 bp
	MtDef4ER R	TTACAGCTCGTCCTTACAATGTG			
14	MtDef4Ec F	ATGGCTCGTTCAGTTCCTTTGG	56	PCR & RT-PCR	401 bp
	MtDef4Ec R	TTAACAATGTGTAGTGCAAAAGC			
15	FMV F	ATTGGGTTCAATCAACAAGG	56	PCR & RT-PCR	542 bp
	FMV R	CTTCAAATGGGAATGAATGC			
16	PR10 F	TGAAACTCACCCAGGGATTG	56	PCR & RT-PCR	523 bp
	PR10 R	TCAGGCGAAAGAGAGAGAGA			
17	OMT F	ACGAAGCCACTGGTAGAGG	56	PCR, RT-PCR & qPCR- copy number	310 bp
	OMT R	CGGTGACGATGTTAGAG			
18	Ver1 F	CGGATTATCCTGACCAGCTC	56	PCR & RT-PCR & qPCR- copy number	330 bp
	Ver1 R	ACCTGCTCATCGGTGAAAGT			
19	FLAV-F	GTCGTCCCCTCTCCGG	60	qRT-PCR-fungal load	100 bp
	FLAV-R	CTGGAAAAAGATTGATTTGCG			
20	FMV CN F	TGCATGTGCTGGAACAGTAGT	60	qPCR- copy number	111 bp
	FMV CN R	TCCAAAGCCTCAACAAGGTCA			
21	GnVP F	ACACCTACAGTTGTCCCAGCTT	60	qPCR- copy number	147 bp
	GnVP R	CTAGGAACCGCTTGAACCCTGT			
22	ADH3 F	GCTTCAAGAGCAGGTCACAAGT	60	RT-PCR & qPCR- copy number	450 bp
	ADH3 R	GAGACATCCTCCTTCGTGCATA			

Table S2. Individual toxin quantification in peanut cotyledons based on ELISA & HPLC following *Aspergillus flavus (AF 11-4)* infection. At least 3-6 biological replicates per event/line were used along with WT control (JL 24), nulls and resistant check (55-437). The individual toxin readings are shown as parts per billion (ppb) +/- SE. Mean data are the average of replicated readings. **Significant at $P < 0.01$, ns indicates non significance, nd indicates non-detectable (LOD = 0.1 ppb), # indicates tested only by ELISA.

**Event**	**AfB$_1$ (ppb)**	**AfB$_2$ (ppb)**	**AfG$_1$(ppb)**	**AfG$_2$(ppb)**
Def4ER 2	7.23±4.34**	0.37±0.01**	nd	nd
Def4ER 5	0.99±0.25**	0.40±0.07**	nd	nd
Def4ER 6	4.48±4.08**	0.36±0.03**	nd	nd
Def4ER 7	1.69±0.92**	0.73±0.16**	nd	nd
Def4Ec 26	0.55±0.03**	0.52±0.06**	nd	nd
Def4Ec 75	0.77±0.24**	0.31±0.04**	nd	nd
Def4Ec 76	0.26±0.02**	0.40±0.03**	nd	nd
Def4Ec 80$^{\#}$	9.135±8.53**	-	-	-
Def4Ec 96	0.91±0.23**	0.65±0.08**	nd	nd
Def4Ec 97	1.12±0.44**	0.80±0.19**	nd	nd
Def1Ec 9	3.00±1.30**	0.67±0.11**	nd	nd
Def1Ec 23	0.74±0.07**	0.74±0.07**	nd	nd
Def1Ec 38$^{\#}$	3.93±2.49**	-	-	-
Def1Ec 46$^{\#}$	5.26±2.11**	-	-	-
Def1Ec 56	2.13±1.75**	0.44±0.06**	nd	nd
OMT 33	6.18±0.62**	0.78±0.16**	nd	nd
OMT 1	0.83±0.11**	0.83±0.11**	nd	nd
OMT 3$^{\#}$	11±4.11**	-	-	-
OMT 14	14.04±10.93**	1.43±0.25**	nd	nd
OMT 16	21.16±4.03**	0.97±0.21**	nd	nd
OMT 17	5.70±2.96**	0.85±0.10**	nd	nd
Ver1 1$^{\#}$	12±3.96**	-	-	-
Ver1 41	2.55±0.55**	1.37±0.62**	nd	nd
Ver1 42	2.75±1.79**	0.68±0.27**	nd	nd
Ver1 6	3.91±1.85**	0.65±0.10**	nd	nd
Null1	3039.15±468.53^{ns}	132.76±16.73*	1.10±0.27^{ns}	2.26±0.14^{ns}
55-437 (RC)	594.20±47.49^{ns}	15.47±1.19*	0.44±0.07^{ns}	0.44±0.07^{ns}
JL 24 (WT)	2085.50±372.29	76.67±13.41	0.69±0.21	1.52±0.43

Table S3. Linear regression analysis between accumulated aflatoxin versus fungal colonization, gene expression of host antioxidative machinery and *A. flavus* aflatoxin cluster genes in OE-Def and HIGS peanut lines.

Variable 1	Lines	Variable 2	Correlation coefficient (r^2)	R value	No. of pairs
B_1 (ppb)	OE-Def	Fungal load	0.624	0.790***	32
		Peanut APX gene expression at 72hpi	0.128	-0.358*	34
		Peanut CAT gene expression at 72 hpi	0.036	-0.190	34
		Peanut SOD gene expression at 72 hpi	0.048	-0.219	34
		aflX gene expression at 72 hpi	0.964	0.982***	12
		aflP gene expression at 72 hpi	0.265	0.514	12
		aflR gene expression at 72 hpi	0.966	0.983***	12
		aflD gene expression at 72 hpi	0.967	0.984***	12
		aflM gene expression at 72 hpi	0.539	0.73443**	12
	HIGS	Fungal load	0.175	0.418	19
		Peanut APX gene expression at 72 hpi	0.053	-0.230	14
		Peanut CAT gene expression at 72 hpi	0.170	-0.412	14
		Peanut SOD gene expression at 72 hpi	0.224	-0.473	14
		aflX gene expression at 72 hpi	0.472	0.687***	38
		aflP gene expression at 72 hpi	0.404	0.636***	38
		aflM gene expression at 72 hpi	0.127	0.356*	38
		aflD gene expression at 72 hpi	0.293	0.541**	38
		aflR gene expression at 72 hpi	0.331	0.575**	38

*, ** and *** reflect statistically significant correlation at $p \leq 0.05$, ≤ 0.01 and ≤ 0.001 respectively.

Table S4. Inheritance studies in segregating peanut OE-Def events during T_1, T_2 and T_3 generations.

Generation	Event#	No. of plants tested	PCR and RT-PCR positives	PCR and RT-PCR negatives	X^2 value
Def4Ec					
T_1	Def4Ec 26	10	6	4	1.20
	Def4Ec 75	6	5	1	0.21
	Def4Ec 76	6	5	1	0.21
	Def4Ec 80	8	5	3	1.20
	Def4Ec 96	5	5	0	1.65
	Def4Ec 97	7	5	2	0.18
T_2	Def4Ec 26-6	6	5	1	0.21
	Def4Ec 75-3	10	8	2	0.13
	Def4Ec 80-1	13	12	1	2.07
	Def4Ec 96-1	20	15	5	0.00
	Def4Ec 96-2	10	8	2	0.2
	Def4Ec 96-3	10	5	5	3.33
	Def4Ec 97-1	12	10	2	0.33
	Def4Ec 97-2	6	4	2	0.21
T_3	Def4Ec 26-6-3	6	4	2	0.21
	Def4Ec 26-6-4	5	3	2	0.60
	Def4Ec 75-3-1	4	3	1	2.00
	Def4Ec 80-1-6	4	3	1	2.00
	Def4Ec 80-1-8	4	4	0	1.30
	Def4Ec 80-1-10	4	2	2	1.30
	Def4Ec 96-1-1	6	3	3	2.00
	Def4Ec 96-1-3	6	3	3	2.00
	Def4Ec 96-1-5	6	3	3	2.00
	Def4Ec 96-1-10	6	4	2	0.21
	Def4Ec 97-1-3	6	5	1	0.21
	Def4Ec 97-1-11	5	4	1	0.06
Def1Ec					
	Def1Ec 3	9	8	1	0.93
T_1	Def1Ec 9	4	2	2	1.30
	Def1Ec 23	8	5	3	1.20
	Def1Ec 38	3	2	1	0.11
	Def1Ec 46	8	5	3	1.20
	Def1Ec 56	8	4	4	2.00
T_2	Def1Ec 3-3	5	5	0	1.67

	Def1Ec 3-6	4	4	0	1.33
	Def1Ec 3-7	1	1	0	1
	Def1Ec 3-10	2	2	0	0.67
	Def1Ec 9-3	6	4	2	0.21
	Def1Ec 23-5	8	7	1	0.62
	Def1Ec 38-1	10	5	5	3.33
	Def1Ec 46-9	7	7	0	2.33
	Def1Ec 56-8	10	9	1	1.2
T_3	Def1Ec 9-3-4	6	5	1	0.21
	Def1Ec 9-3-6	6	6	0	1.30
	Def1Ec 23-5-2	12	10	2	0.33
	Def1Ec 46-9-7	6	4	2	0.21
	Def1Ec 56-8-7	4	4	0	1.30
	Def1Ec 56-8-10	6	4	2	0.21
Def4ER					
T_1	Def4ER 2	10	6	4	1.20
	Def4ER 5	3	2	1	0.1
	Def4ER 6	10	8	2	0.13
T_2	Def4ER 2-1	17	14	3	0.49
	Def4ER 2-8	15	14	1	2.69
	Def4ER 5-1	21	12	9	3.57
	Def4ER 6-7	10	9	1	1.20
	Def4ER 6-10	12	8	4	0.44
	Def4ER 7-3	10	6	4	1.2
	Def4ER 7-4	10	7	3	0.13
	Def4ER 7-5	13	7	6	3.1
	Def4ER 7-6	17	10	7	2.37
T_3	Def4ER 2-1-2	7	5	2	0.18
	Def4ER 2-1-5	7	5	2	0.18
	Def4ER 2-8-6	6	4	2	0.21
	Def4ER 2-8-13	7	5	2	0.18
	Def4ER 5-1-21	6	4	2	0.21
	Def4ER 6-7-2	6	5	1	0.21
	Def4ER 6-7-10	6	4	2	0.21
	Def4ER 6-10-8	6	5	1	0.21

* χ^2 value at 0.05% probability at 1 df is 3.84. Calculated values below 3.84 were non-significant, and the samples fit for 3: 1 segregation ratio.

Table S5. Inheritance studies in segregating peanut HIGS lines during T_1, T_2 and T_3 generations.

Generation	Event#	No. ofplants tested	PCR and RT-PCR positives	PCR and RT-PCR negatives	X^2 value
OMT					
T_1	OMT 1	5	5	0	1.67
	OMT 2	4	2	2	1.33
	OMT 3	6	4	2	0.22
	OMT 7	2	2	0	0.67
	OMT 14	1	1	0	0.33
	OMT 16	2	2	0	0.67
	OMT 17	4	3	1	0.00
	OMT 33	4	2	2	1.33
T_2	OMT 1-1	13	10	3	0.03
	OMT 14-1	11	10	1	1.48
	OMT 15-2	5	3	2	0.60
	OMT 16-1	15	9	6	1.80
T_3	OMT 1-1-11	12	8	4	0.44
	OMT 16-1-2	6	5	1	0.21
	OMT 16-1-6	6	4	2	0.21
	OMT 16-1-15	2	2	0	0.67
Ver 1					
T_1	Ver1 1	2	2	0	0.67
	Ver1 2	8	6	2	0.00
	Ver1 3	7	6	1	0.43
	Ver1 6	2	2	0	0.67
	Ver1 41	4	3	1	0.00
	Ver1 42	4	2	2	1.33
T_2	Ver1 2-3	15	9	6	1.80
	Ver1 3-2	14	10	4	0.09
	Ver1 3-3	10	8	2	0.13
	Ver1 6-1	10	6	4	1.20
	Ver1 6-2	8	6	2	0.00
T_3	Ver1 2-3-1	6	4	2	0.21
	Ver1 2-3-8	6	5	1	0.21
	Ver1 3-3-5	8	6	2	0.00
	Ver1 3-3-7	10	8	2	0.13
	Ver1 3-3-8	4	4	0	1.30
	Ver1 6-2-4	14	12	2	0.85
	Ver1 6-2-9	4	3	1	2.00

* χ^2 value at 0.05% probability at 1 df is 3.84. Calculated values below 3.84 were non-significant, and the samples fit for 3: 1 segregation ratio

Table S6. Overall list of selected lines used in the study and analyses applied to each throughout.

Primary transformant #	Aflatoxin detection (by ELISA and HPLC)	Gene expression studies			Copy number and inheritance	Events advanced
		Defensin genes	Aflatoxin pathway genes	Antioxidants		
Def1Ec 3	✓				✓	✓
Def1Ec 9	✓					✓
Def1Ec 23	✓	✓		✓	✓	✓
Def1Ec 38	✓	✓		✓		✓
Def1Ec 46	✓					✓
Def1Ec 56	✓					✓
Def4Ec 26	✓	✓	✓	✓	✓	✓
Def4Ec 75	✓					✓
Def4Ec 76	✓					✓
Def4Ec 80	✓				✓	✓
Def4Ec 96	✓	✓	✓	✓	✓	✓
Def4Ec 97	✓	✓	✓	✓	✓	✓
Def4ER 2	✓					✓
Def4ER 5	✓					✓
Def4ER 6	✓	✓	✓	✓	✓	✓
Def4ER 7	✓	✓	✓	✓	✓	✓
OMT 1	✓	NA			✓	✓
OMT 2	✓	NA				✓
OMT 3	✓	NA				✓
OMT 14	✓	NA	✓	✓	✓	✓
OMT 16	✓	NA	✓	✓	✓	✓
OMT 17	✓	NA				✓
OMT 33	✓	NA				✓
Ver1 1	✓	NA				
Ver1 2	✓	NA			✓	✓
Ver1 3	✓	NA			✓	✓
Ver1 6	✓	NA	✓	✓	✓	✓
Ver1 41	✓	NA				✓
Ver1 42	✓	NA				✓

Note: NA: Not Applicable
